# Osteoinductive Properties of Autologous Dentin: An Ex Vivo Study on Extracted Teeth

**DOI:** 10.3390/jfb15060162

**Published:** 2024-06-12

**Authors:** Giulia Mazzucchi, Alessia Mariano, Giorgio Serafini, Luca Lamazza, Anna Scotto d’Abusco, Alberto De Biase, Marco Lollobrigida

**Affiliations:** 1 Department of Oral and Maxillofacial Sciences, Sapienza University of Rome, Via Caserta 6, 00161 Rome, Italy; giulia.mazzucchi@uniroma1.it (G.M.); giorgio.serafini@uniroma1.it (G.S.); luca.lamazza@uniroma1.it (L.L.); marco.lollobrigida@uniroma1.it (M.L.); 2 Department of Biochemical Sciences “Alessandro Rossi Fanelli”, Sapienza University of Rome, Piazzale Aldo Moro 5, 00185 Rome, Italy; alessia.mariano@uniroma1.it (A.M.); anna.scottodabusco@uniroma1.it (A.S.d.)

**Keywords:** biomaterials, osteoinductive properties, autologous dentin graft, Smart Dentin Grinder^®^

## Abstract

Over the last decades, a variety of biomaterials, ranging from synthetic products to autologous and heterologous grafts, have been recommended to conserve and regenerate bone tissue after tooth extraction. We conducted a biochemical study on ground extracted teeth that aimed to evaluate the osteoinductive and osteoconductive potential of dentin by assessing the releases of bone morphogenetic protein (BMP-2), osteocalcin (OC) and osteonectin (ON) over time (24 h, 10 days and 28 days). Twenty-six patients, who required the extraction of nonrestorable teeth, were enrolled in the study according to the inclusion criteria, as follows: thirteen young patients 18 to 49 years of age (UNDER 50), and thirteen patients of 50 to 70 years (OVER 50); a total of twenty-six teeth were extracted, ground and analyzed by enzyme-linked immunosorbent assays (ELISA). All ground teeth released BMP-2, OC and ON at each time point; no differences were observed between the UNDER-50 and OVER-50 patients. The results of the study support the use of autologous dentin as osteoinductive material for bone regeneration procedures, irrespective of patients’ ages.

## 1. Introduction

Over the past few decades, regenerative medicine has gained significant attention in the dental field. Guided bone regeneration (GBR) in dentistry traces its roots to the 1980s, evolving from principles used in periodontics [1]. Initially, the focus was on using barrier membranes to facilitate bone growth by preventing soft tissue invasion into bone defects. However, a significant milestone in the advancement of GBR was the development and use of bone substitutes, which have dramatically enhanced the effectiveness and applications of this technique. Bone substitutes were introduced as a solution to the limitations of autogenous bone grafts, which required harvesting bone from a patient’s own body, often resulting in additional surgery and morbidity. The early bone substitutes included allografts, derived from donor bone, and xenografts, sourced from other species, such as bovine bone [2,3,4,5,6]. These materials provided a scaffold for new bone growth, but their success is limited by factors like the inert nature and varying resorption rates. To overcome such limitations, the integration of growth factors and biologics into bone substitutes has been proposed [7]. By incorporating elements such as bone morphogenetic proteins (BMPs) and other biomolecules, these bone substitutes not only serve as scaffolds but also actively promote and accelerate the bone healing process [8]. However, biofunctionalizing bone substitutes is costly and challenging to apply in most clinical situations.

Graft materials should have several ideal properties; they should be biocompatible, resorbable, osteoconductive, osteoinductive, easy to use and cost effective [9]. Autologous bone presents the majority of these characteristics [10,11,12,13,14,15] but needs a donor site, resulting in increased morbidity. Moreover, the reduced or null cell viability due to the harvesting procedure and, in some cases, the high resorption rate, discourages the use of autologous bone in most cases [16]. Other biomaterials with different rates of reabsorption have been suggested but, in most cases, such materials have only osteoconductive properties [7,17].

In recent years, dentine has been proposed as an alternative graft material for bone regeneration to conventional bone substitutes and autologous bone [18,19,20,21]. The use of dentin as an autologous graft in the form of a demineralized dentin matrix (DDM) has emerged because its biological composition closely resembles that of alveolar bone [22,23,24]. Several similarities exist between teeth and bone, including embryological origin, both deriving from neural crest cells, and structural similarities, though with some differences [25,26,27,28]. Alveolar bone comprises approximately 65% inorganic and 25% organic materials. Conversely, dentin consists of 70–75% inorganic and 20% organic components. In addition, both dentin and bone contain type I collagen (90%), and noncollagenous proteins (10%) consisting of osteonectin (ON), dentin sialoprotein (DSP), bone sialoprotein (BSP), osteocalcin (OC), and bone morphogenetic protein (BMP), which are essential for the formation and mineralization of the bone matrix [29,30,31,32,33].

Osteocalcin is produced by osteoblasts and is involved in several biologic processes, including the regulation of bone matrix mineralization and the formation of hydroxyapatite crystals. It also plays a central role in cell adhesion and signaling, facilitating the adhesion of osteoblasts to the bone matrix, promoting the formation and repair of dental tissue and acting as a growth and differentiation factor for osteoblasts [34,35].

Osteonectin is another noncollagenous protein of the bone matrix, which is produced by osteoblasts and odontoblasts. Osteonectin is contained in both dentin and enamel, with a higher concentration in dentin. It binds to collagen, providing strength and flexibility to the dental tissue. Osteonectin also plays a role in the mineralization of dentin and enamel, promoting the formation of hydroxyapatite crystals [34,35].

BMPs have also been identified in the human dentin matrix after demineralization [33]. BMPs have osteoinductive capacity, promoting the differentiation of mesenchymal stem cells into osteoblasts [36]. Although bone-derived BMPs and dentin-derived BMPs have different biochemical structures, they have similar functions in regulating the development and repair of osseous and dental tissues [23,32,33].

DDM primarily functions as a scaffold to support cell migration (osteoconduction) but may also elicit an osteoinductive action due to the presence of dentinal BMP-2 and other molecules. It has been observed, however, that mineral components of dentin could trap the BMPs limiting their bioavailability. Demineralization has been suggested as a solution for the release of a higher amount of growth factors and noncollagenous proteins [37]. Both demineralized bone matrix (DBM) and demineralized dentin matrix (DDM) retain type I collagen, growth factors and BMP-2 [18,36].

Considering the abovementioned biological properties of DDM, extracted teeth were first utilized as autologous graft materials for regenerative procedures [38]. Autologous dentine can be inserted into the postextractive or atrophic sites of the same patient without causing inflammation or rejection and is gradually resorbed and replaced by new bone [39,40].

To further support the use of ground teeth as osteoinductive graft material, the aim of this study was to investigate the release over time of BMP-2, OC and ON from ground extracted teeth.

## 2. Materials and Methods

### 2.1. Patient Recruitment, Inclusion and Exclusion Criteria

Twenty-six patients who required the extraction of nonrestorable teeth were included in the study, as follows: 13 patients aged between 18 and 49 years (UNDER 50 group), and 13 patients aged between 50 and 70 years (OVER 50 group).

The donor patients were enrolled according to the following inclusion criteria:

- Patients in need of dental extraction due to severe caries, coronal and/or radicular fracture, nontreatable periapical lesion, grade 2 or higher mobility, untreatable periodontal defects;
- Patients in need of extraction of impacted mandibular third molars with partial/total bony/osteo-mucosal inclusion, following recurrent abscesses, pericoronitis, severe caries affecting the third molar, severe caries affecting the adjacent second molar that cannot be otherwise treated without prior extraction of the third molar, distal periodontal defects of the adjacent second molar, root resorption affecting the adjacent second molar, dysplastic lesions affecting the mandibular third molar;
- Patients willing and able to provide written informed consent;
- Nonsmoker or light smoker (less than 10 cigarettes per day);
- Absence of contraindications to materials and anesthesia;
- Good overall health.

Patients who required extraction of teeth with root filling material were excluded from the study.

### 2.2. Ethics Statement

The study followed the principles of the Declaration of Helsinki in relation to the research work involving human species, and it received approval from the Ethics Committee of Policlinico Umberto I of Rome, Italy (protocol code: 5456/2019, 25 July 2019). Patients were enrolled in the study after signing a written informed consent form.

### 2.3. Grinding of Teeth

Immediately after the extraction, teeth were removed of enamel, cement, remaining calculus, caries, and periodontal ligament using a tungsten carbide bur and then dried with an air syringe. Obtained teeth were then ground for 3 s and sorted for 10 s using a dedicated device (Smart Dentin Grinder™, KometaBio Inc., Cresskill, NJ, USA). After grinding, the granules produced were sorted into the following two compartments with drawers: an upper compartment and a lower compartment. Granules between 300 and 1200 μm in diameter with a porosity of 2–14 μm accumulated in the upper compartment, while granules smaller than 300 μm accumulated in the lower compartment [41,42]. A 0.5 g sample of granules was taken from the upper drawer compartment and placed in a sterile Dappen dish, soaked with 0.5 M NaOH and 20% ethanol solution for 10 min to dissolve organic remains from the dentinal tubules and rinsed two times (3 min each) with phosphate-buffered saline solution (PBS). The granules were then immersed in 2 mL PBS solution and stored at 4 °C. The conditioned PBS was then collected and analyzed at 24 h (T1), 10 days (T2) and 28 days (T3) for the quantification of the BMP-2, osteocalcin (OC) and osteonectin (ON).

### 2.4. ELISA Tests

The amount of BMP-2, OC and ON released in the PBS was determined using enzyme-linked immunosorbent assay kits (Fine Test ELISA, Fine Biotech Co., Ltd., Wuhan, China), according to the manufacturer’s instructions. Briefly, 100 μL of conditioned PBS was added to the wells and analyzed in duplicate. The plate was static incubated for 90 min at 37 °C. Then, the plate was washed twice, and 100 μL of biotin-labeled antibody was added to each well and incubated for 60 min at 37 °C. After incubation, the plate was washed three times and incubated with 100 μL of HRP–streptavidin conjugate for 30 min at 37 °C. Then, the plate was washed five times and 90 μL of tetramethylbenzidine substrate was added and incubated for 15 min at 37 °C to develop the blue color. Finally, 50 μL of an acid solution was added to turn the color to yellow. The optical density (O.D.) absorbance was measured at 450 nm by a microplate reader (NB-12-0035, NeoBiotech, Holden, MA, USA).

### 2.5. Statistical Analysis

The statistical analysis was performed using a nonparametric one-way ANOVA (Kruskal–Wallis test) and post hoc Mann–Whitney *U* (MWU) test (adjusted by Bonferroni correction for multiple tests) and applied on the original data to obtain a comparison among groups at each time point. The data were plotted in histograms using Prism 5.0 software (GraphPad Software, San Diego, CA, USA).

## 3. Results and Discussion

**Figure 1:**
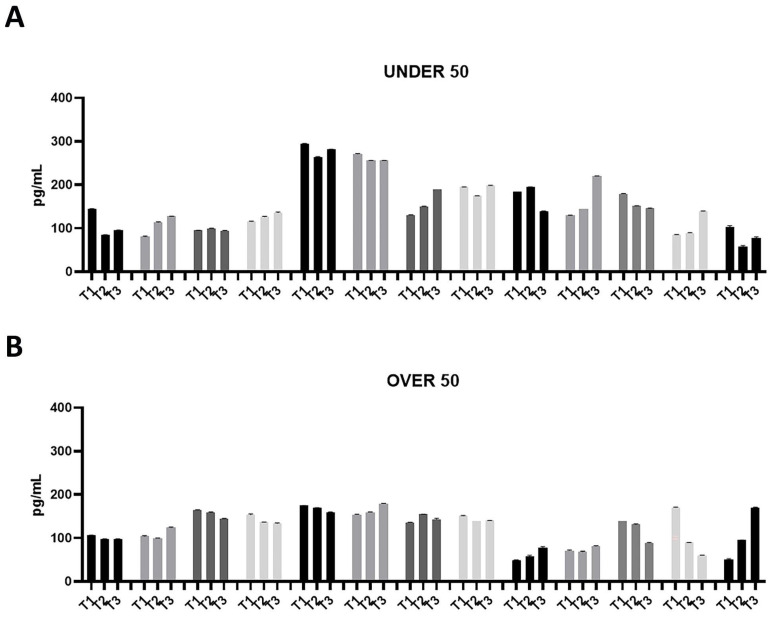
Bone morphogenetic protein (BMP)-2 releases over time in the UNDER 50 and OVER 50 patients. The BMP-2 release in the ground teeth was determined at T1 (24 h), T2 (10 days) and T3 (28 days) by an ELISA test: (**A**) UNDER 50 group with 13 patients; (**B**) OVER 50 group with 13 patients. The data from each patient are grouped and reported as different grey shades (T1, T2 and T3). The results are expressed as the mean ± standard deviation of the data obtained by duplicate analyses, performed by Prism 5.0 software (GraphPad).

**Figure 2:**
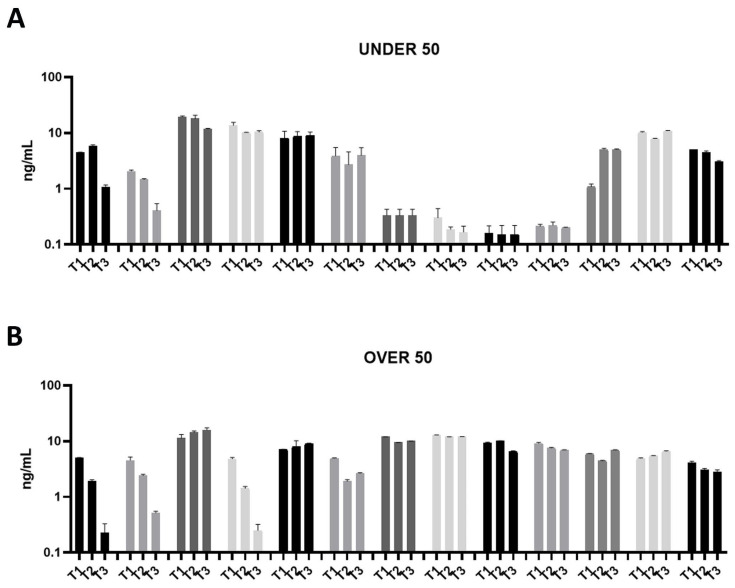
Osteocalcin (OC) releases over time in the UNDER 50 and OVER 50 patients. The OC release in the ground teeth was determined at T1 (24 h), T2 (10 days) and T3 (28 days) by an ELISA test: (**A**) UNDER 50 group with 13 patients; (**B**) OVER 50 group with 13 patients. The data from each patient are grouped and reported as different grey shades (T1, T2 and T3). The results are expressed as mean ± Standard Deviation of data obtained by duplicate analyses, performed by Prism 5.0 software (GraphPad).

**Figure 3:**
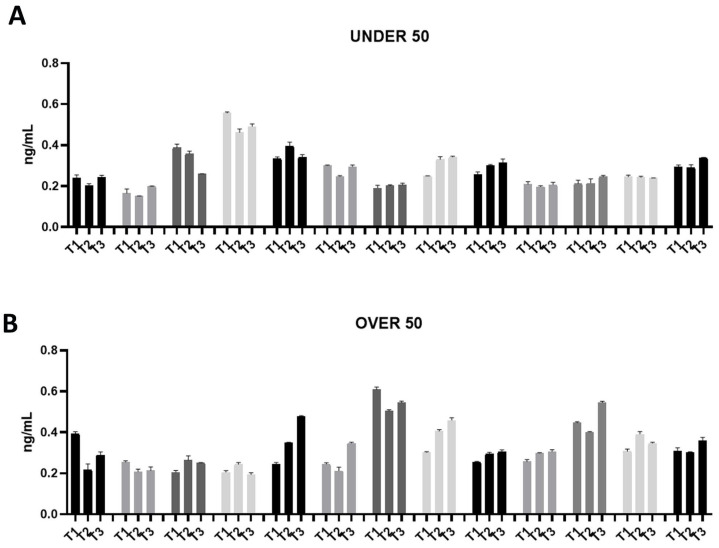
Osteonectin (ON) releases over time in the UNDER 50 and OVER 50 patients. The ON release in the ground teeth was determined at T1 (24 h), T2 (10 days) and T3 (28 days) by an ELISA test: (**A**) UNDER 50 group with 13 patients; (**B**) OVER 50 group with 13 patients. The data from each patient are grouped and reported as different shades of grey (T1, T2 and T3). The results are expressed as the mean ± standard deviation of data obtained by duplicate analyses, performed using Prism 5.0 software (GraphPad).

In order to determine the odonto/osteoinductive capacity of the ground teeth, the release of three factors (i.e., BMP-2, OC and ON), which play important roles in matrix organization, mineralization and bone formation, was evaluated. The amount of BMP-2, OC and ON released in PBS was measured at 24 h (T1), 10 days (T2) and 28 days (T3) after extraction. As shown in Figure 1, Figure 2 and Figure 3, all growth factors had already been released after 24 h and continued until day 28. An interindividual difference in the amount of factor release was observed without statistically significant differences.

The patients in the UNDER 50 group showed a release of BMP-2 ≤ 200 pg/mL, at three time points; only two patients showed a release of about 300 pg/mL (Figure 1A). All patients in the OVER 50 group showed a release ≤ 200 pg/mL (Figure 1B). In most samples, the release was constant over time; only one patient showed an increasing release from T1 to T3 (Figure 1B).

All of the analyzed ground teeth had higher productions of OC compared to BMP-2. The BMP-2 was released in a concentration of picograms, whereas the OC was released in a concentration of nanograms. Most specifically, the OVER 50 group showed comparable releases of around 10 ng/mL, while in the UNDER 50 group a more interindividual variability is reported (Figure 2A,B). In the UNDER 50 group, four patients showed a very low release of OC, approximately 0.5 ng/mL, at three time points (Figure 2A). In the OVER 50 group, all samples showed a constant release at all analyzed times, except for three samples that exhibited decreases over time, in particular after 28 days, with a final concentration lower than 0.5 ng/mL (Figure 2B).

The ON release was assessed in a range of approximately 0.2–0.6 ng/mL in all the samples from both groups (Figure 3A,B). Moreover, the release remained constant over time. Compared to OC, ON did not show an interindividual variability.

**Figure 4:**
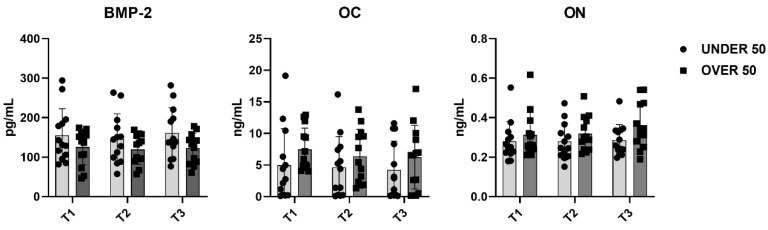
The average release of bone morphogenetic protein-2 (BMP-2), osteocalcin (OC) and osteonectin (ON) reported for all analyzed times (T1: 24 h; T2: 10 days; and T3: 28 days) in the UNDER 50 and OVER 50 groups. The results are expressed as the mean ± standard deviation of data obtained by duplicate analyses, performed using Prism 5.0 software (GraphPad). The statistical analyses were performed using a nonparametric one-way ANOVA (Kruskal–Wallis test) and post hoc Mann–Whitney *U* (MWU) test (adjusted by Bonferroni correction for multiple tests).

To further verify whether statistical differences between the UNDER 50 and OVER 50 group could be observed, a comparative analysis was performed. The medium releases of the three growth factors for each analyzed time are reported in Figure 4, with light grey representing the UNDER 50 group and dark grey the OVER 50 group. BMP-2 and ON did not show any difference between the groups and over time. The release of the OC factor was higher in the OVER 50 patients compared to the UNDER 50 subjects, for all analyzed times. The main difference was observed for T1, even if the differences were not statistically significant (T1, *p* = 0.06; T2, *p* = 0.22; T3, *p* = 0.36).

**Figure 5:**
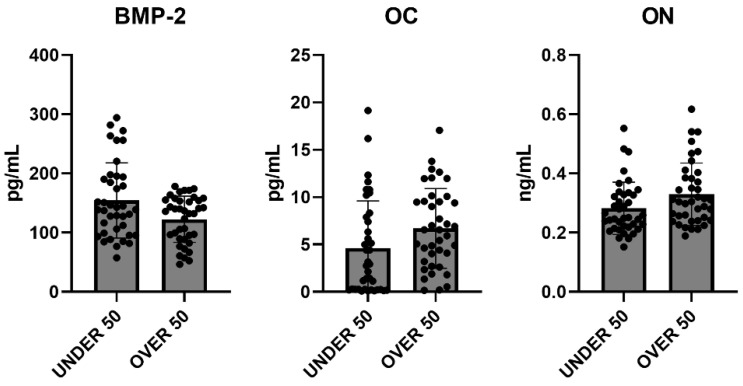
The average cumulative release of bone morphogenetic protein-2 (BMP-2), osteocalcin (OC) and osteonectin (ON) considering all samples from the UNDER 50 and OVER 50 groups at all analyzed times. The results are expressed as the mean ± standard deviation of data obtained by duplicate analyses, performed using Prism 5.0 software (GraphPad). The statistical analysis was performed using a nonparametric one-way ANOVA (Kruskal–Wallis test) and post hoc Mann–Whitney *U* (MWU) test (adjusted by Bonferroni correction for multiple tests).

To evaluate the overall release of the three factors after 28 days, a comparative analysis was carried out. The average differences among patients and between the UNDER 50 and OVER 50 groups were not statistically significant. The averages of the released amounts at the three time points and for all samples, divided according to the two groups, UNDER 50 and OVER 50, are reported in Figure 5. As described in the above analysis, the BMP-2 and ON showed comparable releases in the two groups, whereas only the OC showed a difference. The release was higher in the OVER 50 group, even if the absence of statistical significance was confirmed.

The last decades have been characterized by a great interest in biomaterials for bone regeneration. After tooth extraction, bone remodeling invariably leads to a loss of bone volume. A variety of biomaterials, spanning from synthetic products to autologous or heterologous substitutes, are currently used after the tooth extraction to minimize this phenomenon [43].

It is accepted that the ideal graft material should be osteogenic, osteoinductive and osteoconductive at the same time [9]. From this perspective, only autologous bone meets all of these requirements [14,44]. Other widely used graft materials, such as heterologous bone or alloplastic materials grafts, can have excellent osteoconductive properties even if without osteoinduction [45].

In this scenario, the possibility of using extracted teeth as an autologous graft material in the form of demineralized dentin matrix (DDM) has come to the fore. The first reason for using DDM in bone regeneration is represented by their similarities in embryonic origin and chemical composition [46].

A review article published by Zhang et al. [47] compared DDM to other graft materials highlighting some peculiarities. In addition to the biocompatibility, the authors highlight the higher acceptance of DDM by patients compared to allografts from cadavers. Another advantage that also contributes to patients’ acceptance is represented by the availability of dentin, eliminating the need for bone harvesting from other sites. The authors also conclude that autologous dentin is as effective as autologous bone in bone regeneration.

Like autologous bone, DDM is cost effective, potentially reducing the overall treatment cost compared to other bone substitutes.

In this study, we confirmed the release of BMP-2, OC and ON from ground dentin. Similar to other bone substitutes, demineralized dentin matrix (DDM) primarily functions as a scaffold [37]. However, in addition to its osteoconductive properties, the presence and release of BMP-2 endow it with osteoinductive capabilities, promoting the differentiation of mesenchymal stem cells into osteoblasts and subsequent bone matrix deposition. Furthermore, no significant differences in growth factor release were observed between the UNDER 50 and OVER 50 groups. Initially, we hypothesized that the potential of dentin to provide growth factors might decrease with age. This hypothesis was based on the relative increase in the amounts of secondary and tertiary dentin compared to primary dentin over a lifetime. However, our results suggest that age does not affect the regenerative potential of dentin. This can be explained by the fact that secondary dentin forms as a secondary layer along the entire pulp–dentin border throughout life, without replacing the primary dentin, and tertiary dentin is produced only in localized areas in response to harmful stimuli. Consequently, teeth, and specifically dentin, can be regarded as a reservoir of molecules beneficial for regeneration, maintaining their stability throughout an individual’s lifetime. This finding further supports the use of autologous dentin, regardless of a patient’s age, whenever possible.

Different devices are available on the market for grinding extracted teeth. A study conducted in 2023 by Dłucik R et al. [48] compared the efficacy of three DDMs obtained from different medical devices on the market (Tooth Transformer Smart, Smart Dentin Grinder and BonMaker). Despite some variations in structures and chemical compositions of the granules, the authors concluded that all of the devices could generate particles with adequate characteristics for bone regeneration. The present study was conducted using a patented medical device specifically designed to convert extracted teeth into DDM. Differently from other devices, the overall preparation procedure takes only a few minutes [19], making it particularly suitable for clinical practice. Moreover, since no weak acids have been used in the postground treatment, only a partial demineralization could be expected. Given the high mineral content of teeth, demineralizing protocols have been recently recommended to preserve autologous growth factors, including BMP-2, and to facilitate their sustained release over time [19,49]. Demineralizing protocols typically involve the use of weak acids, such as hydrochloric acid (HCl) or ethylenediaminetetraacetic acid (EDTA). This process removes the mineral content, exposing the organic matrix, which primarily consists of collagen and other noncollagenic molecules, including BMPs. Demineralized tooth granules may exhibit enhanced osteoinductive properties due to the exposure of growth factors such as BMPs compared to nondemineralized granules, although no specific literature data currently support this claim. Nonetheless, it has been observed that the demineralization process can render dentin remarkably similar to natural bone in terms of surface chemical composition [50].

While the biochemical study confirms the osteoinductive potential of dentin granules, the initial hypothesis regarding age-related influence was not supported, since the statistical analysis revealed no correlation between interindividual variability and patient’s age. These findings call for alternative explanations. Firstly, factors related to bone metabolism might influence the mineral and organic content of the teeth at the time of their development; secondly, unknown aspects of patients’ medical history or metabolism could play a role even after tooth development. Further studies involving larger populations should more thoroughly investigate various patient-related factors, such as the osteometabolic profile, which may influence the content and release of growth factors from dentin.

This study has some limitations, including the relatively small size of the study population and the lack of prior laboratory data on patients’ bone metabolism, which would help investigate factors that might influence the content of growth factors in dentin. Additionally, the inclusion of different teeth with varying eruption ages may have introduced variability into the results.

While recent research has concentrated on characterizing dentin particles based on various grinding and postgrinding processes, future studies need to explore the biological activity of dentin grafts. This aspect is crucial as it distinguishes dentin grafts from other graft materials, such as xenografts and alloplastic bone substitutes. Once the potential of dentin grafts is fully elucidated, extracted teeth will justifiably be regarded as a valuable biological resource for bone regeneration procedures. For the same reason, it will be fundamental to investigate the possibility of storing ground teeth for future use with the same patient.

## 4. Conclusions

The results of this study indicate that BMP-2, OC and ON are effectively released by dentine granules over time. The statistical analysis demonstrated that the release of growth factors is not affected by age, though some variability exists among the patients, particularly for the OC release. These findings confirm DDM as an alternative to traditional biomaterials for bone regeneration procedures. Considering the limitations of this study, further research with larger sample sizes is warranted to confirm the findings and explore potential relationships with other patient-related factors.

## Data Availability

The data presented in this study are available upon request from the corresponding author.
